# Development and Evaluation of a Manganese and Iron Food Frequency Questionnaire for Pediatrics

**DOI:** 10.3390/ijerph14091060

**Published:** 2017-09-14

**Authors:** Frida B Zipkin, Grace A Falciglia, Pierce Kuhnell, Erin N Haynes

**Affiliations:** 1 Department of Environmental Health, University of Cincinnati College of Medicine, 160 Panzeca Way, Kettering Laboratory, Cincinnati, OH 45267, USA; fzipkin@gmail.com; 2 Department of Nutritional Sciences, University of Cincinnati, 3202 Eden Avenue, Cincinnati, OH 45267, USA; grace.falciglia@gmail.com; 3 Department of Epidemiology and Biostatistics, Cincinnati Children’s Hospital Medical Center, 3333 Burnet Avenue, Cincinnati, OH 45229, USA; pierce.kuhnell@cchmc.org

**Keywords:** manganese, iron, toxicity, diet, questionnaire, development, evaluation, Appalachian, pediatric, population

## Abstract

Manganese (Mn) is an essential nutrient, but overexposure can lead to neurotoxicity. Given the essentiality of Mn in the diet, particularly during children’s growth and development, it is imperative to quantify dietary Mn intake in populations that may be exposed to industrial sources of Mn. Dietary absorption of Mn is inversely associated with iron (Fe) stores, yet there is currently no food frequency questionnaire (FFQ) to assess dietary Mn and Fe intake. The study objective was to develop and evaluate the validity of a FFQ to measure dietary Mn and Fe intake in pediatrics by comparing the estimated intakes of Mn and Fe with biomarkers: Mn in blood and hair and Fe in serum. This study utilized a subset of the Communities Actively Researching Exposure Study (CARES) population residing in Guernsey County, Ohio. Dietary Mn was not correlated with either blood or hair Mn; however, dietary Mn and serum ferritin were significantly correlated, with a correlation coefficient of 0.51, *p* < 0.01. Moreover, dietary Fe and serum ferritin were also significantly correlated, with a correlation coefficient of 0.51, *p* < 0.01. This FFQ is a valid measurement tool for Fe intake as measured by serum ferritin; however, Mn intake did not correlate with either blood or hair Mn.

## 1. Introduction

Manganese (Mn) is an essential nutrient for humans and is required in trace amounts to maintain cellular processes in the body [1]. While Mn deficiency is extremely rare in humans, toxicity due to overexposure is more prevalent [2]. Ingested Mn is primarily controlled by tight homeostatic absorption and excretion regulation. However, the intestinal absorption of Mn has been shown to increase during low iron (Fe) stores, which may increase the risk of Mn accumulation in the brain and affect children’s neurodevelopment [3,4,5].

Recent studies have reported adverse effects of Mn exposure on cognitive [6,7,8,9,10,11], motor [12,13], and behavioral [14,15] functions in children. Unfortunately, only one of these studies [6] examined the contribution of dietary Mn measured by a 50-item Food Frequency Questionnaire (FFQ), a questionnaire that had been developed by experts in the field of nutrition, but it found no association with Mn concentration in children’s hair. In addition, there is currently no validated FFQ to assess dietary Mn intake in children.

Optimally, the evaluation of the FFQ is done against a noninvasive ‘gold standard’ biomarker for the nutrient of interest. In the absence of data supporting the use of a specific biomarker for Mn, multiple biomarkers for exposure have been considered in research involving children. Blood and hair are the most commonly collected Mn biological indicators in recent pediatric population studies [6,7,9,10,12,13,14]. The concentration of Mn in blood is expected to indicate a recent exposure (days before sampling) while the concentration of Mn in hair is expected to indicate exposure over several months or years [11,16].

From all the tools known to assess the usual dietary intake of nutrients in large-scale studies, the FFQ is the most practical, economical, and frequently used method [17]. The primary aim of this study was to develop and evaluate the validity of a FFQ to measure dietary Mn and Fe intake in a rural pediatric population by comparing the estimated intakes of Mn and Fe with corresponding Mn and Fe biomarkers (Mn in blood and hair and Fe in serum ferritin).

## 2. Methods

### 2.1. Study Design and Population

Children were recruited to participate in the Communities Actively Researching Exposure Study (CARES) if they were seven, eight, or nine years of age and resided in Washington County, Ohio, Wood County, West Virginia, or Guernsey County, Ohio throughout their life with no plans to move for at least one year. In addition, their biological mother must have resided in the catchment area during her pregnancy with the index child. Families were recruited for participation in CARES using a volunteer sampling strategy. Recruitment postcards were sent home with children through schools, and advertisements were aired on local radio and printed in local newspapers. Children were excluded if they did not meet the eligibility criteria or had a health condition that impeded their ability to participate in the behavioral assessment testing such as a significant visual, auditory, or motor impairment. The University of Cincinnati Institutional Review Board approved this study. All parents and children signed an informed consent form, and the children also signed an informed assent form.

This study utilized a subset (*n* = 81) of the CARES cohort residing in Guernsey County, Ohio. Guernsey County has no known ambient source of Mn exposure [18]. The families were enrolled in the study from October 2008 to March 2013.

### 2.2. Study Procedure

Trained personnel administered the semi-quantitative FFQ. The study personnel assisted parents in this process and verified the responses for food item completion. Standardized measuring cups and spoons, visual aids, and food models were used to aid food portion size estimation. A calendar indicating the last seven days of food intake was also used to help focus the parent or legal guardian on the precise time frame. The study data were entered into the Research Electronic Data Capture (REDCap), a secure web-based system for data collection [19]. Biospecimens were collected from participants during the same study visit.

### 2.3. Food Frequency Questionnaire and Dietary Data Collection and Analysis

**Figure 1:**
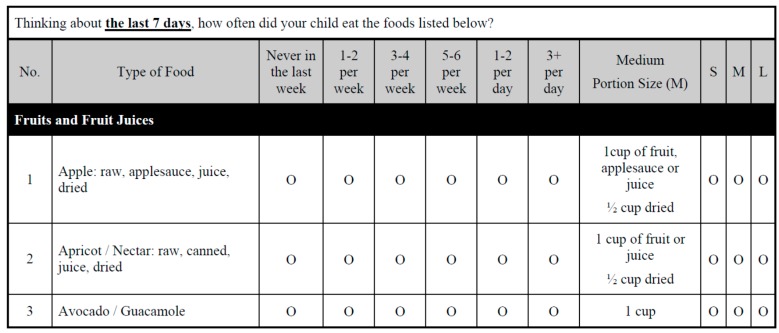
Example of the Manganese and Iron Food Frequency Questionnaire heading, first food group, and first three food item.

The FFQ was developed to capture the diverse diet of the rural Appalachian pediatric population, with emphasis on foods rich in Mn and Fe (Figure 1). The FFQ was constructed following the Dietary Guidelines for Americans published by the U.S. Government in 2005 [20,21]. A cover page of the FFQ contained detailed instructions on how to complete the questionnaire. The FFQ listed 115 food items and provided five blank rows for ‘other foods’ not captured in the questionnaire. Foods with high Mn and Fe contents were identified using the United States Department of Agriculture (USDA) Agricultural Research Service, National Nutrient Database for Standard Reference, Nutrient Lists—Report by Nutrients: Manganese and Iron [22]. Foods typical to the Appalachian culture were obtained from additional sources [23]. The full Mn and Fe FFQ is provided in the Supplementary Materials.

Several strategies were used to develop a comprehensively constructed FFQ. This questionnaire was organized by five food groups well known to the general public [20,21]. Each food group had a list of foods for that group. Foods that share similar nutrient contents, e.g., cake/cookies/cupcakes, were combined into one group in a coherent and easy to understand format in order to increase the number of foods to be included in a FFQ of reasonable length. Several options (raw, frozen, canned, etc.) for perishable food were available for each item to reflect seasonal variations and availability differences during the year.

A medium portion size for each food item was obtained from published U.S. Department of Agriculture data [20,21]. The brand names of cooked and ready-to-eat cereals were requested. The energy, Mn, and Fe content of each medium serving size food item were obtained using the 2005 Nutrient Data System for Research database developed by the University of Minnesota.

Parents or legal guardians were asked to indicate how frequently their child consumed the selected food item during the previous seven days by checking one of six categories, which ranged from ‘never in the last week’ to ‘3+ per day’. For each food item, a medium portion size was specified, and participants could mark the portion size as small (½ medium portion size), medium, or large (1 ½ medium portion size). The frequency of intake was multiplied by the usual portion size reported to calculate the portion per week for each food item of the FFQ. The kilocalories of energy, grams of fat, carbohydrate, and protein and milligrams of Mn and Fe per medium portion size were multiplied by the portion per week for each line item in order to determine the weekly dietary intake of each line item for each child. Finally, the average daily amounts were calculated from the total weekly amounts by dividing by seven.

Data from completed questionnaires were entered in duplicate and compared for errors. The following criteria were employed to classify a questionnaire as complete. A FFQ was considered complete when only four or fewer food items, excluding the ‘other food’ category, were left blank (≥95% completed). If a food item was left blank on the FFQ, it was coded as ‘never in the last week’ (not consumed). A FFQ was considered incomplete, and therefore data was not entered, when five or more food items, excluding the ‘other food’ items, had been left blank (<95% completed).

### 2.4. Biological Data Collection and Analysis

All biospecimens were collected by a trained phlebotomist. Venous whole-blood specimens were collected from the antecubital vein in 3 mL purple top (K2EDTA) tubes certified by the analyzing laboratory for trace element analysis. Specimens were refrigerated at 5 °C until they were shipped to the Laboratory of Inorganic and Nuclear Chemistry at the New York State Department of Health’s (NYS DOH) Wadsworth Center for analysis in Albany, New York. The blood specimens were analyzed for Mn using graphite furnace atomic absorption spectrometry (GFAAS, PerkinElmer^®^ Model 4100 ZL) equipped with a transversely-heated graphite atomizer and a longitudinal Zeeman background correction system (PerkinElmer^®^ Life and Analytical Sciences, Shelton, WA, USA) using the previously described method and quality-control measures [24,25]. The Method Detection Limit (MDL) for Mn in blood was 1.5 µg/L.

Serum ferritin was analyzed using a Clinical Laboratory Improvement Amendments (CLIA)-certified clinical laboratory at Marietta Memorial Hospital. After the samples were spun at 3000 rpm for 10 min, they were loaded into the Abbott Architect ci8200 Integrated System Chemistry analyzer, where the test for serum ferritin was performed. The results were electronically transmitted to the University of Cincinnati.

Approximately 20 strands of hair were collected from the occipital region, cut with ceramic scissors as close to the scalp as possible. It was ensured that the hair was at least 1 cm in length for analysis. Long hair was trimmed to 6 cm and taped towards the non-scalp-side end of the hair shaft onto an index card with an arrow pointing in the direction of the scalp end on the index card. The card with the taped hair sample was placed into a pre-labeled envelope and stored at room temperature until it was shipped to the Channing Trace Metals Laboratory, Brigham and Women’s Hospital, Harvard School of Public Health for analysis in Boston, Massachusetts. The samples were first washed in a 1% TritonTM X-100 solution and then digested using concentrated HNO_3_. Acid digestates were then analyzed by Inductively coupled plasma mass spectrometry (ICP-MS) using the previously described methods [26]. The method detection limit (MDL) for Mn in hair was <2 ng/g.

### 2.5. Statistical Analysis

Of the 81 Guernsey County CARES participants, 78 participants had a completed questionnaire according to the criteria described above. The daily estimated intake of energy was obtained for these questionnaires. Participants with daily estimates of their energy intake below 500 or higher than 5000 kcal were excluded from all analyses following established precedents from previous FFQ validation studies involving children and adolescents [27,28,29]. Thus, 29 participants were excluded from the analysis. Therefore, the final numbers of participants in the analysis were 52 children.

Mn concentrations in hair and whole blood were included in the matrix as internal biological markers of Mn exposure. Serum ferritin closely reflects Fe stores [30,31,32,33] and was included in the matrix as an internal biomarker for Fe exposure.

Descriptive statistics were conducted. Pearson’s correlation coefficients matrix was used to investigate the association between dietary Mn, dietary Fe, and biological data. Statistical analysis of the data obtained from the FFQ was restricted to energy and five nutrients (fat, carbohydrate, protein, Mn, and Fe). The data were examined for normality prior to analysis and were log transformed when necessary. The statistical significance was set at *p* < 0.05. All analyses were performed using SAS statistical software, version 9.2 (SAS Institute Inc., Cary, NC, USA).

## 3. Results

The demographic characteristics and biological measurers of the CARES participants from Guernsey County children are summarized in Table 1. The majority of the participants were Caucasian (88%, *n* = 71). The mean age of the participants was 8.3 ± 0.9 years; 8.3 ± 0.8 years for girls and 8.4 ± 1.0 years for boys. The mean hair Mn concentration was 549.6 ± 2.6 ng/g, and the mean blood Mn concentration was 9.1 ± 1.3 µg/L. The mean serum ferritin concentration was 35.3 ± 1.7 µg/mL; 36.7 ± 1.6 µg/mL and 30.1 ± 1.7 µg/mL for females and males, respectively. The demographic characteristics were similar for the excluded and included participants.

Table 2 presents geometric mean and geometric standard deviation (GM, GSD, respectively), and the Pearson correlation coefficients matrix for dietary Mn, dietary Fe, and biomarkers for Mn and Fe. The mean blood Mn concentration was 9.10 ± 1.30 µg/L, ranging from 5.3 µg/L to 17.4 µg/L. The mean hair Mn concentration was 0.58 ± 2.67 µg/g and ranged from 0.09 µg/g to 4.2 µg/g. The blood Mn and the hair Mn were significantly correlated, with a correlation coefficient of 0.41, *p* < 0.05.

The daily means for dietary Mn and Fe intakes were 2.39 ± 1.81 mg and 12.81 ± 1.74 mg, respectively. The daily means were slightly above the Adequate Intake (AI) for both minerals but were not statistically significant [1]. Dietary Mn and dietary Fe were significantly correlated, with a correlation coefficient of 0.88, *p <* 0.001. Many good sources of dietary Mn are also good sources of dietary Fe, especially non-heme Fe [22]. Dietary Mn and dietary Fe were not correlated with either blood or hair Mn in this subsample. 

The mean serum ferritin concentration was 34.33 ± 1.71 µg/mL, ranging from 13 µg/mL to 89 µg/mL, which is within the normal range for age and gender [33]. Dietary Fe and serum ferritin were significantly correlated, with a correlation coefficient of 0.51, *p* < 0.01. Furthermore, dietary Mn and serum ferritin were also significantly correlated, with correlation coefficient of 0.51, *p* < 0.01.

## 4. Discussion

In this subgroup of the CARES cohort, correlation coefficients of 0.51 were observed for both dietary Mn and Fe, calculated from the FFQ developed for this study and the concentrations of ferritin in serum. The results of this study suggest that the proposed FFQ provides valid estimates of dietary Fe exposure. Furthermore, serum ferritin appeared to be a suitable biomarker for Fe dietary exposure.

The 120-item FFQ was developed to capture the weekly Mn and Fe intake with emphasis on foods rich in these minerals. Bouchard et al. had previously developed a similar but shorter FFQ [6]. This 50-item questionnaire, not previously validated, was used to obtain monthly estimates of dietary Mn intake in children exposed to various Mn levels in their drinking water. To our knowledge, this is the second study that used Mn biomarkers to investigate the validity of a FFQ to measure dietary Mn exposure in a pediatric population. 

In the present evaluation study, correlation coefficients of −0.21 and 0.03 were obtained for dietary Mn intake compared to blood and hair Mn, respectively. The results indicated that the dietary Mn intake estimates did not correlate with the blood or hair estimates. Similarly, Bouchard et al. reported that Mn intake from diet was not in association with hair Mn [6]. Despite the fact that food consumption is the primary source of Mn intake for the general population [34], there has been little to no research on the influence of ingested Mn in the diet, particularly in the pediatric population. Physiological changes in Mn gastrointestinal absorption, liver metabolism, and biliary and pancreatic excretion are known to occur in order to maintain constant tissue Mn concentrations [4,35,36,37,38,39,40]. Thus, the fraction of ingested Mn retained by the body is tightly regulated in order to maintain normal tissue Mn concentrations under different dietary conditions. Additionally, no reliable biomarkers of dietary Mn exposure are known [2,41,42,43]. 

Several factors influence Mn absorption. The absorption of Mn from a meal decreases as the Fe content in the meal increases. Thus, intestinal absorption of Mn is increased during low Fe status [4]. In the CARES subsample, all participants had serum ferritin levels within the typical range for this age [33]. The metabolic interaction of the two metals has been described in previous studies [4,44,45]. In addition, the concentration of Mn in the diet is known to influence the amount of gastrointestinal Mn absorption, as well as its excretion via bile. A high Mn dietary intake usually results in decreased gastrointestinal absorption, as well as increased biliary and pancreatic excretion of Mn [4,36,37,39,46,47]. Furthermore, the presence of phytates, oxalates, tannins, and other dietary constituents are known to decrease the absorption of Mn [48,49,50,51]. Additionally, age and gender is known to influence the gastrointestinal tract absorption of Mn [52]. Males have reduced gastrointestinal Mn absorption compared to females.

The present study has several limitations. One of the limitations was the small number of participants that participated in the FFQ validation study. Another limitation was the restriction on the number of food items that can be included in the food frequency. Mn and Fe are widely distributed in nearly all foods, although not in equal content. To overcome this challenge, a comprehensive focus semi-quantitative FFQ developed for this study emphasized foods that are good sources of both minerals, which have a greater impact on Mn and Fe dietary intake. Measurement error and bias with respect to exactly what was consumed, preparation methods, and portion sizes, especially in dishes with various ingredients, is almost inevitable. Also, the dietary data collected relied on the individual’s memory of the foods consumed over the prior seven days [53]. Another potential limitation is the lack of water Mn data; however, we did analyze 20 households that participated in the subanalysis for water Mn. The geometric mean of the water in Mn was 0.00105 ng/mL (std, 0.00037 ng/mL). The mean is well below the secondary drinking water standard set by the US EPA of 0.05 mg/L and thereby does not represent an important source of exposure for participants in this study.

In conclusion, the FFQ developed for this study is a valid instrument due to the strong association between dietary Fe and Fe measures. This FFQ could be used to process data in a large-scale epidemiological study; however, given the small number of participants in this study, further development of the FFQ would likely improve its use in larger studies. The implication of this study underscores the need to search for optimal biomarkers of dietary Mn in children, since they are considered more vulnerable to the effects of environmental toxicants than adults [54,55,56,57]. In the future, studies using this FFQ could further evaluate estimates of dietary Mn intake and test additional Mn biomarkers of exposure. The FFQ could be further developed and used in advance assessments of both the health benefits of Mn and its toxicity in children.

## 5. Conclusions

This FFQ provides reliable estimates on Fe intake in children, which may help to assess the levels of these minerals in populations at risk. Future studies are needed to reevaluate the estimates of dietary Mn intake and other Mn biomarkers.

## Figures and Tables

**Table 1:** Demographic characteristics and biological measurers of children enrolled in CARES ^a^ from Guernsey County, OH, USA.

Characteristics	Cohort (n = 81)	Female (n = 28)	Male (n = 53)
**Child Measures**	mean ± SD ^b^		
Age (years)	8.3 ± 0.9	8.3 ± 0.8	8.4 ± 1.0
Race/ethnicity	n (%)		
Caucasian	71 (88)	28 (100)	43 (81)
Black/African American	5 (6)	0 (0)	5 (9)
Other	5 (6)	0 (0)	5 (9)
**Biological Measurers**	GM ^c^ ± GSD ^d^		
Hair Mn (ng/g), n = 70	549.6 ± 2.6	488.1 ± 2.6	656.8 ± 2.7
Blood Mn (μg/L), n = 65	9.1 ± 1.3	8.8 ± 1.2	9.7 ± 1.3
Serum Ferritin (μg/mL), n = 64	34.3 ± 1.7	36.7 ± 1.6	30.1 ± 1.7

^a^ CARES = Community Actively Researching Exposure Study; ^b^ SD = standard deviation; ^c^ GM = geometric mean; ^d^ GSD = geometric standard deviation.

**Table 2:** Geometric mean, *geometric standard deviation*, and Pearson correlation coefficients matrix for dietary and biomarkers variables of Mn and Fe in CARES ^a^ from Guernsey County, OH, USA.

Variable	GM ^b^ (GSD ^c^)	Dietary Mn	Dietary Fe	Blood Mn	Hair Mn	Serum Ferritin
Dietary Mn (mg/d), n = 52	2.39 (1.81)	1.00				
Dietary Fe (mg/d), n = 52	12.81 (1.74)	0.88 ***	1.00			
		52				
Blood Mn (μg/L), n = 40	9.10 (1.30)	−0.21	−0.04	1.00		
		40	40			
Hair Mn (μg/g), n = 47	0.58 (2.67)	0.03	−0.04	0.41 *	1.00	
		47	47	36		
Serum Ferritin (μg/mL), n = 39	34.33 (1.71)	0.51 **	0.51 **	−0.12	−0.06	1.00
		39	39	39	35	

^a^ CARES = Community Actively Researching Exposure Study; ^b^ GM = *geometric mean*; ^c^ GSD = *geometric standard deviation*; * *p* < 0.05; ** *p* < 0.01; *** *p* < 0.001.

## Supplementary Materials

The following are available online at www.mdpi.com/1660-4601/14/9/1060/s1, Title: Manganese and Iron Food Frequency Questionnaire.
