# Supplementary material for: Development and Evaluation of a Manganese and Iron Food Frequency Questionnaire for Pediatrics
Source: Int J Environ Res Public Health. 2017 Sep 14;14(9):1060. doi: 10.3390/ijerph14091060 (PMC5615597; doi:10.3390/ijerph14091060)
Supplement: Supplementary file 1 [file ijerph-14-01060-s001.pdf]

Study ID: __-__-__-_
Date: ___/___/___

Date of birth: ____/____/____

Interviewer Name: ____ (Interviewer Code: ____ __-_)

FOOD QUESTIONNAIRE

Questionnaire refers to what you ate over the last seven days

This Food Questionnaire asks you about the type and frequency (how often) your child ate the foods listed below **during the last 7 days**. Since we recognize it may be hard to remember every detail of your child's diet, please do the best you can. We truly appreciate your effort. This information will be helpful in understanding the role of diet in your child's health.

This Food Questionnaire takes about 15 minutes.

Please follow the instructions:

- For each food item, mark **ONLY ONE** response.
- Serving sizes: **M** (medium) = 1 portion size
  S (small) = ½ medium portion size
  L (large) = 1 ½ medium portion size
- Mark your response as accurate as you can.
- Estimate if you are not sure.
- Use a **NO. 2 PENCIL** only.
- Do not use ink or ballpoint pen.
- Darken in circle completely:

⇒ CORRECT MARK ●

⇒ INCORRECT MARK ✓ X ⊘

- Erase cleanly any marks you wish to change.
- Do not make any stray marks on this form.

THANK YOU FOR COMPLETING THIS FOOD QUESTIONNAIRE!

Thinking about **the last 7 days**, how often did your child eat the foods listed below?

No.	Type of Food	Never in the last week	1-2 per week	3-4 per week	5-6 per week	1-2 per day	3+ per day	Medium Portion Size (M)	S	M	L
**Fruits and Fruit Juices**											
1	Apple: raw, applesauce, juice, dried	O	O	O	O	O	O	1cup of fruit, applesauce or juice ½ cup dried	O	O	O
2	Apricot / Nectar: raw, canned, juice, dried	O	O	O	O	O	O	1 cup of fruit or juice ½ cup dried	O	O	O
3	Avocado / Guacamole	O	O	O	O	O	O	1 cup	O	O	O
4	Banana: raw, cooked, dehydrated slices	O	O	O	O	O	O	1 cup of fruit ½ cup dehydrated	O	O	O
5	Blackberries: raw, frozen, canned, cooked, frozen	O	O	O	O	O	O	1 cup	O	O	O
6	Blueberries: raw, frozen, canned, cooked, frozen	O	O	O	O	O	O	1 cup	O	O	O
7	Grapefruit: raw, canned, juice	O	O	O	O	O	O	1 cup of fruit or juice	O	O	O
8	Grapes: raw, juice, raisins	O	O	O	O	O	O	1 cup of fruit or juice ½ cup raisins	O	O	O
9	Melon (all varieties)	O	O	O	O	O	O	1 cup	O	O	O
10	Olives	O	O	O	O	O	O	1 cup	O	O	O
11	Orange / Tangerine: raw, canned, juice	O	O	O	O	O	O	1 cup of fruit or juice	O	O	O
12	Peach / Nectarine: raw, canned, juice, dried	O	O	O	O	O	O	1 cup of fruit or juice ½ cup dried	O	O	O
13	Pear: raw, canned, juice, dried	O	O	O	O	O	O	1 cup of fruit or juice ½ cup dried	O	O	O
14	Pineapple: raw, canned, juice, dried	O	O	O	O	O	O	1 cup of fruit or juice ½ cup dried	O	O	O
15	Plum / Prune: raw, canned, juice	O	O	O	O	O	O	1 cup of fruit or juice ½ cup prunes	O	O	O
16	Raspberries: raw, frozen, canned	O	O	O	O	O	O	1 cup	O	O	O
17	Strawberries: raw, frozen, canned	O	O	O	O	O	O	1 cup	O	O	O
18	Watermelon	O	O	O	O	O	O	1 cup	O	O	O
19	Other: …………………………………………..…	O	O	O	O	O	O		O	O	O

No.	Type of Food	Never in the last week	1-2 per week	3-4 per week	5-6 per week	1-2 per day	3+ per day	Medium Portion Size (M)	S	M	L
**Vegetables**											
20	Asparagus: raw, cooked, canned	O	O	O	O	O	O	1 cup	O	O	O
21	Broccoli: raw, cooked	O	O	O	O	O	O	1 cup raw<br>½ cup cooked	O	O	O
22	Brussels Sprouts	O	O	O	O	O	O	1 cup	O	O	O
23	Butternut Squash	O	O	O	O	O	O	1 cup	O	O	O
24	Cabbage (all varieties): raw, cooked	O	O	O	O	O	O	1 cup	O	O	O
25	Carrots: raw, cooked	O	O	O	O	O	O	1 cup raw<br>¾ cup cooked	O	O	O
26	Cauliflower: raw, cooked	O	O	O	O	O	O	1 cup raw<br>½ cup cooked	O	O	O
27	Celery	O	O	O	O	O	O	1 cup	O	O	O
28	Collards: raw, cooked	O	O	O	O	O	O	1 cup raw<br>½ cup cooked	O	O	O
29	Corn: cream style, cooked, canned, on cob	O	O	O	O	O	O	1 cup<br>1 medium corn on cob	O	O	O
30	Cucumber: raw, pickled	O	O	O	O	O	O	1 cup	O	O	O
31	Dandelion Greens: raw, cooked	O	O	O	O	O	O	1 cup raw<br>½ cup cooked	O	O	O
32	Eggplant	O	O	O	O	O	O	1 cup	O	O	O
33	Green, String, Snap, Wax Beans or Snow Peas	O	O	O	O	O	O	1 cup	O	O	O
34	Green Zucchini	O	O	O	O	O	O	½ cup	O	O	O
35	Lettuce (all varieties)	O	O	O	O	O	O	1 cup	O	O	O
36	Mustard / Turnip Greens: raw, cooked	O	O	O	O	O	O	1 cup raw<br>½ cup cooked	O	O	O
37	Mushroom (all varieties): raw, cooked, canned, pickled	O	O	O	O	O	O	1 cup raw<br>½ cup cooked or dehydrated	O	O	O
38	Okra: raw, cooked	O	O	O	O	O	O	1 cup	O	O	O
39	Onion / Green Onions: raw, cooked, dehydrates	O	O	O	O	O	O	1 cup raw<br>½ cup cooked or dehydrated	O	O	O
40	Peas: raw, cooked, canned	O	O	O	O	O	O	1 cup	O	O	O
41	Pepper (all varieties except red / orange / yellow bell pepper): raw, cooked, pickled	O	O	O	O	O	O	1 cup raw<br>½ cup cooked	O	O	O
42	Potato (all varieties): cooked, mashed, French fries	O	O	O	O	O	O	1 medium<br>1 cup	O	O	O
43	Red / Orange / Yellow Bell Pepper: raw, cooked, pickled	O	O	O	O	O	O	1 cup raw<br>½ cup cooked	O	O	O

DO NOT DUPLICATE

No.	Type of Food	Never in the last week	1-2 per week	3-4 per week	5-6 per week	1-2 per day	3+ per day	Medium Portion Size (M)	S	M	L
**Vegetables**											
44	Radish	O	O	O	O	O	O	1 cup	O	O	O
45	Spinach: raw, cooked, canned	O	O	O	O	O	O	1 cup raw ½ cup cooked	O	O	O
46	Sweet Potato/Yam	O	O	O	O	O	O	1 large ¾ cup	O	O	O
47	Tomato (all varieties): raw, canned, cooked, salsa, sauce, soup, juice	O	O	O	O	O	O	1 cup	O	O	O
48	Turnip greens	O	O	O	O	O	O	1 cup	O	O	O
49	Winter Squash / Pumpkin (all varieties except butternut squash): cooked, canned	O	O	O	O	O	O	1 cup	O	O	O
50	Yellow Zucchini: raw, cooked	O	O	O	O	O	O	1 cup	O	O	O
51	Other: ……………………………………..…	O	O	O	O	O	O		O	O	O
**Bread, Cereal, Rice, and Pasta**											
52	Bread	O	O	O	O	O	O	1 slice	O	O	O
53	Bagel, Pita Bread, English Muffin, Hotdog or Hamburger Bun	O	O	O	O	O	O	½ medium piece (1 ounce)	O	O	O
54	Bulgur	O	O	O	O	O	O	½ cup	O	O	O
55	Barley, dry	O	O	O	O	O	O	½ cup	O	O	O
56	Cake / Cookies / Cupcake	O	O	O	O	O	O	½ cup	O	O	O
57	Cereal or Granola Bar	O	O	O	O	O	O	1 bar	O	O	O
58	Cooked cereal: brand name …………………………………………… ……………………………………………	O	O	O	O	O	O	½ cup	O	O	O
59	Ready-to-eat cereal: brand name …………………………………………… ……………………………………………	O	O	O	O	O	O	1 cup	O	O	O
60	Pretzel / Chex Mix / Corn or Bagel Chips	O	O	O	O	O	O	1 cup	O	O	O
61	Crackers	O	O	O	O	O	O	6 crackers	O	O	O
62	Doughnut / Danish/Pastry / Pop tart	O	O	O	O	O	O	½ medium piece	O	O	O
63	Muffin / Biscuit	O	O	O	O	O	O	1 small	O	O	O
64	Pancake / Waffles / French Toast	O	O	O	O	O	O	1 medium 1 slice	O	O	O
65	Pasta: noodles, spaghetti, macaroni	O	O	O	O	O	O	½ cup	O	O	O

DO NOT DUPLICATE

No.	Type of Food	Never in the last week	1-2 per week	3-4 per week	5-6 per week	1-2 per day	3+ per day	Medium Portion Size (M)	S	M	L
**Bread, Cereal, Rice, and Pasta**											
66	Oat bran, raw	O	O	O	O	O	O	½ cup	O	O	O
67	Pie crust	O	O	O	O	O	O	1/8 slice of a 8-inch pie	O	O	O
68	Pizza crust	O	O	O	O	O	O	1 slice of a 12" pizza	O	O	O
69	Rice, white or brown	O	O	O	O	O	O	½ cup	O	O	O
70	Tortilla / Taco shell	O	O	O	O	O	O	1 small	O	O	O
71	Other: ………………………………………..…	O	O	O	O	O	O		O	O	O
**Meat, Poultry, Fish, Dry Beans, Eggs, and Nuts**											
72	Bacon regular	O	O	O	O	O	O	3 slice	O	O	O
73	Beans / Lentil / Chickpeas (all variety): cooked, canned	O	O	O	O	O	O	¼ cup	O	O	O
74	Beef including ground	O	O	O	O	O	O	1 ounce ¼ cup	O	O	O
75	Chicken including ground	O	O	O	O	O	O	1 ounce ¼ cup	O	O	O
76	Deer including ground	O	O	O	O	O	O	1 ounce ¼ cup	O	O	O
77	Duck / Goose	O	O	O	O	O	O	1 ounce ¼ cup	O	O	O
78	Egg	O	O	O	O	O	O	1 egg 2 egg white	O	O	O
79	Fish / Tuna fish: all varieties, fresh, frozen or canned	O	O	O	O	O	O	1 ounce ¼ cup	O	O	O
80	Hot dog / Sausage	O	O	O	O	O	O	1 medium 1 ounce ¼ cup	O	O	O
81	Lamb including ground	O	O	O	O	O	O	1 ounce ¼ cup	O	O	O
82	Liver, heart, kidney, tongue, brain	O	O	O	O	O	O	1 ounce ¼ cup	O	O	O
83	Lunch meats such as salami, bologna, chicken, turkey or ham	O	O	O	O	O	O	1 ounce ¼ cup 1slice	O	O	O
84	Nuts / Seeds / Peanuts (in tablespoons, Tbls)	O	O	O	O	O	O	2 Tbls (½ ounce)	O	O	O
85	Peanut Butter	O	O	O	O	O	O	1 tablespoon	O	O	O
86	Pork / Ham including ground	O	O	O	O	O	O	1 ounce ¼ cup	O	O	O
87	Rabbit / Hare / Squirrels	O	O	O	O	O	O	1 ounce ¼ cup	O	O	O

DO NOT DUPLICATE

No.	Type of Food	Never in the last week	1-2 per week	3-4 per week	5-6 per week	1-2 per day	3+ per day	Medium Portion Size (M)	S	M	L
**Meat, Poultry, Fish, Dry Beans, Eggs, and Nuts**											
88	Shellfish / Clam / Shrimp	O	O	O	O	O	O	1 ounce<br>¼ cup	O	O	O
89	Soybean Products / Meatless Foods: tofu, chili, hamburger, nuggets, meatballs, sausages	O	O	O	O	O	O	1 ounce<br>¼ cup	O	O	O
90	Turkey including ground or turkey bacon	O	O	O	O	O	O	1 ounce<br>¼ cup	O	O	O
91	Veal including ground	O	O	O	O	O	O	1 ounce<br>¼ cup	O	O	O
92	Other: ……………………………………..…	O	O	O	O	O	O		O	O	O
**Milk, Yogurt, and Cheese**											
93	Buttermilk	O	O	O	O	O	O	1 cup	O	O	O
94	Cheese: American, Cheddar or other firm cheese	O	O	O	O	O	O	2 slices<br>1.5 ounces	O	O	O
95	Cottage cheese	O	O	O	O	O	O	1 cup	O	O	O
96	Cream Cheese	O	O	O	O	O	O	1 cup	O	O	O
97	Custard / Pudding	O	O	O	O	O	O	1 cup	O	O	O
98	Evaporated or Condensed Milk	O	O	O	O	O	O	1 cup	O	O	O
99	Flavored Milk: chocolate, vanilla, strawberry or other flavor	O	O	O	O	O	O	1 cup	O	O	O
100	Frozen Yogurt / Sherbet / Iced Milk	O	O	O	O	O	O	1 cup<br>2 scoop	O	O	O
101	Goat Milk or Cheese	O	O	O	O	O	O	1 cup	O	O	O
102	Ice Cream	O	O	O	O	O	O	1 cup<br>2 scoop	O	O	O
103	Milk	O	O	O	O	O	O	1 cup	O	O	O
104	Milk Shake	O	O	O	O	O	O	1 cup	O	O	O
105	Sour Cream	O	O	O	O	O	O	1 cup	O	O	O
106	Soy Milk / Veggie Slices	O	O	O	O	O	O	1 cup<br>2 slices	O	O	O
107	Whipping / Whipped Cream	O	O	O	O	O	O	1 cup	O	O	O
108	Yogurt	O	O	O	O	O	O	1 cup	O	O	O

DO NOT DUPLICATE

No.	Type of Food	Never in the last week	1-2 per week	3-4 per week	5-6 per week	1-2 per day	3+ per day	Medium Portion Size (M)	S	M	L
**Miscellaneous Foods**											
109	Butter, Margarine, Oil	O	O	O	O	O	O	2 pats<br>2 teaspoons	O	O	O
110	Mayonnaise / Salad Dressing	O	O	O	O	O	O	1 tablespoon	O	O	O
111	Potato Chips / Cheese Puffs	O	O	O	O	O	O	1 cup	O	O	O
112	Gravy, Sauces (all varieties except tomato sauce)	O	O	O	O	O	O	¼ cup	O	O	O
113	Milk Chocolate, Candy Bar	O	O	O	O	O	O	3 small pieces<br>1 small bar	O	O	O
114	Sugar, Jam, Jelly, Honey, Syrup	O	O	O	O	O	O	2 teaspoons	O	O	O
115	Sport, Fruit Flavored, or Soft Drink	O	O	O	O	O	O	1 cup	O	O	O
116	Semisweet / dark chocolate	O	O	O	O	O	O	3 small pieces	O	O	O
117	Tea, instant sweetened with sugar	O	O	O	O	O	O	1 cup	O	O	O
118	Popcorn	O	O	O	O	O	O	3 cups	O	O	O
119	Trail Mix	O	O	O	O	O	O	½ cup	O	O	O
120	Other: ……………………………………..…	O	O	O	O	O	O		O	O	O

* *

Medium Portion Sizes (M) based on definition from U.S. Department of Agriculture, Center for Nutrition Policy and Promotion: MyPyramid.gov (USDA, 2005).

DO NOT DUPLICATE

Overall Diet Questionnaire

1. During the last 7 days, how many times did your child eat foods that contain whole-grains?

☐ 0 – 1 time ☐ 2 – 3 times ☐ 4 – 5 times ☐ 6 – 7 times

2. During the last 7 days, how many times did your child eat fried foods?

☐ 0 – 1 time ☐ 2 – 3 times ☐ 4 – 5 times ☐ 6 – 7 times

3. During the last 7 days, how many times did your child eat in a fast food restaurant?

☐ 0 – 1 time ☐ 2 – 3 times ☐ 4 – 5 times ☐ 6 – 7 times

4. During the last 7 days, how many times did your child eat lean cuts of meat?

☐ 0 – 1 time ☐ 2 – 3 times ☐ 4 – 5 times ☐ 6 – 7 times

5. During the last 7 days, how many times did your child eat or drink low-fat dairy products?

☐ 0 – 1 time ☐ 2 – 3 times ☐ 4 – 5 times ☐ 6 – 7 times

6. During the last 7 days, how many cups of fruit juices did your child drink a day?

☐ 0 – 1 cup ☐ 2 – 3 cups ☐ 4 or more cups

7. During the last 7 days, how many fruits did your child eat?

☐ 0 – 1 cup ☐ 2 – 3 cups ☐ 4 or more cups

8. During the last 7 days, how many cups of vegetables did your child eat per day?

☐ 0 – 1 cup ☐ 2 – 3 cups ☐ 4 or more cups

9. During the last 7 days, were the fruits and vegetables your child ate grown locally?

- <u>Fruits</u> ☐ Yes ☐ No ☐ Don't know
- <u>Vegetables</u> ☐ Yes ☐ No ☐ Don't know

Overall Diet Questionnaire (continue)

10. During the last 7 days, did your child take any multivitamins/vitamins/minerals /supplements?

☐ No ☐ Yes

If yes, complete table. Provide product name and how many tablets/capsules/chewable (see example above).
How much was given to your child each day? (Specify # of tables, gummies, or amount of liquid).

Product Name (Please Specify)	How much was given to your child each day? (specify # of tablets, gummies, or mL of liquid)				
	Tablets	Gummies	Jellies	Liquid	Other
**Multivitamins (e.g.):** **Flintstones Complete with Omega-3**		**2**			
Multivitamins: ……………………………………………………………………					
Vitamins: ……………………………………………………..……………..					
Minerals: ……………………………………………………….……………					
Supplements: ……………………………………………………….………….....					

During the last 7 days, how many times did your child take the multivitamins/vitamins/minerals /supplements?

Multivitamins	☐ 0 – 1 time	☐ 2 – 3 times	☐ 4 – 5 times	☐ 6 – 7 times
Vitamins	☐ 0 – 1 time	☐ 2 – 3 times	☐ 4 – 5 times	☐ 6 – 7 times
Minerals	☐ 0 – 1 time	☐ 2 – 3 times	☐ 4 – 5 times	☐ 6 – 7 times
Supplements	☐ 0 – 1 time	☐ 2 – 3 times	☐ 4 – 5 times	☐ 6 – 7 times

11. During the last 7 days, how often did your child engage in physical activity?

☐ Less than 30 minutes a day ☐ 30 to 60 minutes a day ☐ More than 60 minutes a day
